# Citalopram-Induced Oral Melanotic Macules in a Female Patient and a Review of the Literature

**DOI:** 10.7759/cureus.60889

**Published:** 2024-05-23

**Authors:** Vasileios Zisis, Petros Papadopoulos, Eleftherios Anagnostou, Dimitrios Andreadis, Athanasios Poulopoulos

**Affiliations:** 1 Oral Medicine/Pathology, Aristotle University of Thessaloniki, Thessaloniki, GRC

**Keywords:** psychotropic drugs, drug-induced adverse reaction, citalopram, melanocytic benign lesion, macule

## Abstract

Pigmented lesions in the oral cavity can arise from the accumulation of external substances or internal pigments, resulting in black or brown discoloration. The etiology can be categorized as physiologic, reactive, neoplastic, idiopathic, or indicative of systemic illness. Several systemic drugs have been linked to the development of oral and/or cutaneous pigmentation, either by stimulating the production of melanin or by the accumulation of the drug or its byproducts. The medications most commonly associated with this condition include antimalarials, hormones, oral contraceptives, phenothiazines, chemotherapeutics, amiodarone, minocycline, zidovudine, clofazimine, and ketoconazole. The aim of this case report is to illustrate the drug-induced appearance of multiple melanotic macules in an 89-year-old female patient. The patient was referred to the Department of Oral Medicine and Pathology, School of Dentistry, Aristotle University of Thessaloniki, Greece, complaining of the recent and constant appearance of black spots in her oral cavity. Her medical history revealed a multitude of prescribed drugs, with citalopram being the most recently prescribed one, approximately one year prior to the examination. The clinical examination revealed multiple melanotic macules, on the upper and lower lip as well as on the hard and soft palate. Based on these findings, a biopsy of a melanotic macule of the lip was carried out. The histopathological examination showed that the basal layer of the stratified squamous epithelium exhibited hyperpigmentation (melanin-pigmented basal cells). In addition, scattered melaninophages were noted in lamina propria. Psychotropic drugs associated with cutaneous hyperpigmentation include citalopram. Therefore, our case constitutes an exception since citalopram induced intraoral and perioral, instead of cutaneous, hyperpigmentation.

## Introduction

Pigmented lesions in the oral cavity can arise from the accumulation of external substances or internal pigments, resulting in black or brown discoloration [1]. The etiology can be categorized as physiologic, reactive, neoplastic, idiopathic, or indicative of systemic illness. Although the role of melanocytes in oral mucosa is not fully understood, they are located in the basal layer and are responsible for the production of melanin. This melanin can be absorbed by epithelial cells or released into the underlying connective tissue [2]. Physiologic pigmentation, also known as racial pigmentation, manifests as pigmented spots on the skin that are macular in nature, exhibiting diverse forms and sizes. This condition can be observed in individuals from various ethnic backgrounds [1]. It is more frequently observed in individuals with darker skin and is caused by heightened melanotic activity rather than an increase in the number of melanocytes [3]. Pigmentation is seen in individuals of both genders, at all ages, although its prevalence tends to rise with advancing age and its intensity may be heightened due to variables such as smoking, hormonal fluctuations, and the use of systemic drugs [4]. The pattern and hue of gingival pigmentation vary significantly among individuals of different races, however, it is important to acknowledge the existence of diversity within a particular racial group [1]. Pigmentation can range in hue from pale beige to deep brown to nearly black. Physiologic pigmentation can occur in any part of the mouth, but it is most frequently observed in the attached gingiva and in descending order of occurrence, the buccal mucosa, lips, and palate [4]. With the exception of gingival involvement, the pigmentation typically appears in irregular shapes with less distinct boundaries [5]. The pigmentation on the upper surface of the tongue may affect exclusively the fungiform papillae [1]. Under microscopic examination, physiologic pigmentation reveals heightened melanin concentration in the basal epithelial layer and melanin leakage in the superficial lamina propria [3]. The diagnosis of physiologic pigmentation relies on a thorough clinical examination, and therapy is not required.

Entities that may display comparable characteristics encompass systemic diseases such as Addison's illness [6], Peutz-Jeghers syndrome [7], and Laugier-Hunziker syndrome [8]. The pigmented lesions are typically found in multiple locations, although their appearance can range from mild to striking, even within the same illness [4]. Biopsy findings reveal that in each of the three aforementioned disorders, pigmentation is caused by an elevated amount of melanin in the basal layer [6-8]. Individuals with dark skin are also susceptible to acquiring post-inflammatory pigmentation in the oral cavity, which can be observed in cases of persistent trauma or inflammatory diseases [3,4,6]. A smoker's melanosis constitutes a reactive disorder that causes discoloration of the oral mucosa due to smoking cigarettes, cigars, or pipes [1]. This phenomenon is attributed either to the presence of harmful substances in cigarette smoke or the activation of melanocytes by heat, which triggers the defensive production of melanin [9]. Smoker’s melanosis is encountered in adults, in approximately 21.5-30% of all smoking individuals [1,10]. Smoker's melanosis is most frequently located in the anterior labial mandibular gingiva and may also be observed on the buccal mucosa, lip, hard palate, and tongue [11]. Usually, there are several macules, that vary in color from light brown to brown-black, depending on how long and how often the person has been smoking tobacco [12]. Mucosal pigmentation may arise from the accumulation of external substances, such as dental amalgam, tattoo pigment, or graphite, as well. The most prevalent occurrence among these is the amalgam tattoo, which is observed in 3.3% of the adult population in the United States [13]. This can happen when placing or removing a dental restoration, where small pieces can penetrate the soft tissues. Amalgam tattoo appears as a flat discoloration, which may manifest as gray, blue, or black, exhibiting clear, uneven, or indistinct borders, with a size of 6mm or less [14]. Gingiva and alveolar mucosa are the sites, most commonly involved, followed by the buccal mucosa and the floor of the mouth [14]. The aim of this case report is to illustrate the drug-induced appearance of multiple melanotic macules in an 89-year-old female patient.

## Case presentation

**Figure 1:**
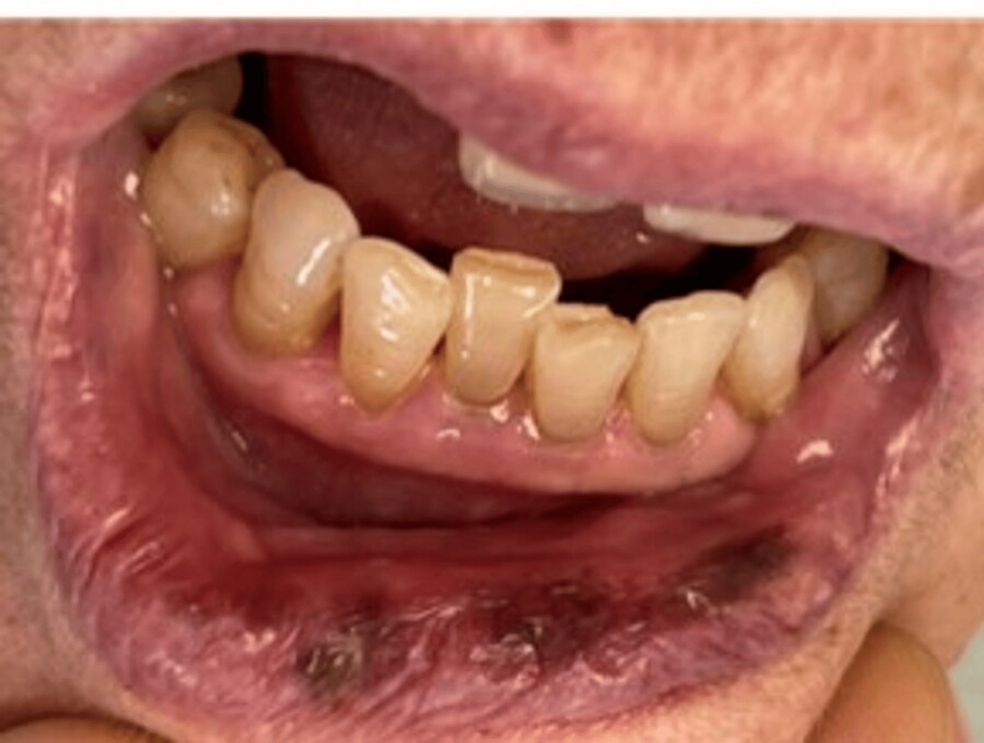
Melanotic macule on the lower lip

**Figure 2:**
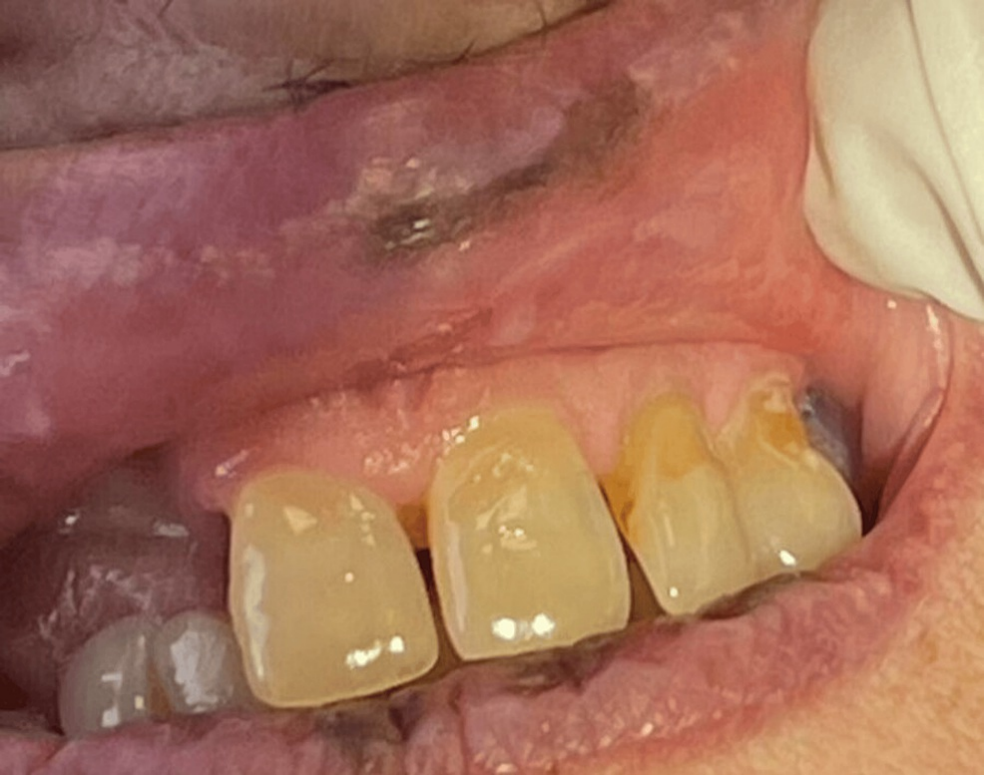
Melanotic macule on the upper lip

**Figure 3:**
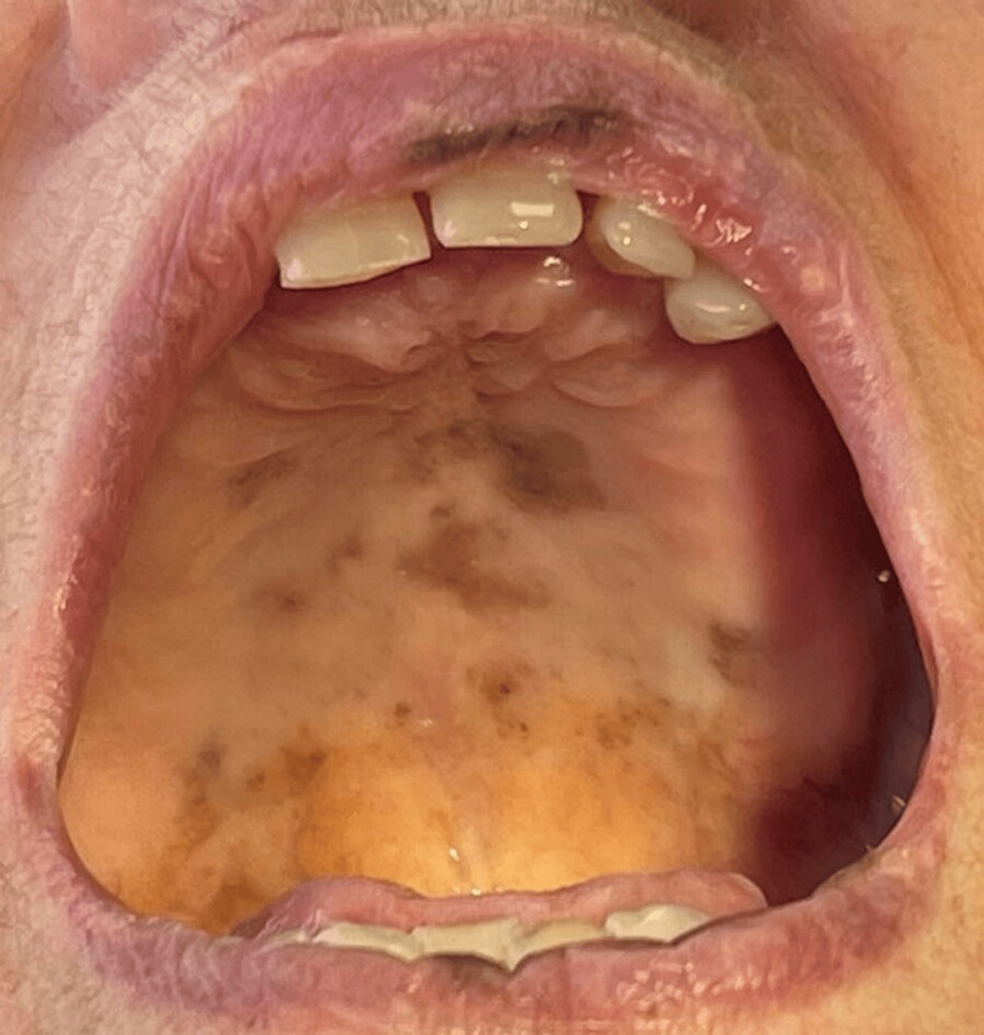
Melanotic macule on the palate

A female patient, 89 years old, was referred to the Department of Oral Medicine and Pathology, School of Dentistry, Aristotle University of Thessaloniki, Greece, complaining of the recent and constant appearance of black spots in her oral cavity. Before the examination, the patient provided written informed consent. This form was approved by the School of Dentistry, Aristotle University of Thessaloniki, and was in accordance with the Helsinki Declaration for Research and Patient Ethics. Subsequently, the patient was examined thoroughly. Her medical history revealed hypertension, hypercholesterolemia, osteoporosis, mild depression, and the concurrent uptake of allopurinol, irbesartan, hydrochlorothiazide, pravastatin sodium, denosumab, bromazepam, and citalopram. Regarding the onset of each drug administration, the patient mentioned that citalopram was the most recently prescribed one, approximately one year prior to the examination. The clinical examination revealed multiple melanotic macules, on the upper and lower lip as well as on the hard and soft palate (Figures 1-3).

**Figure 4:**
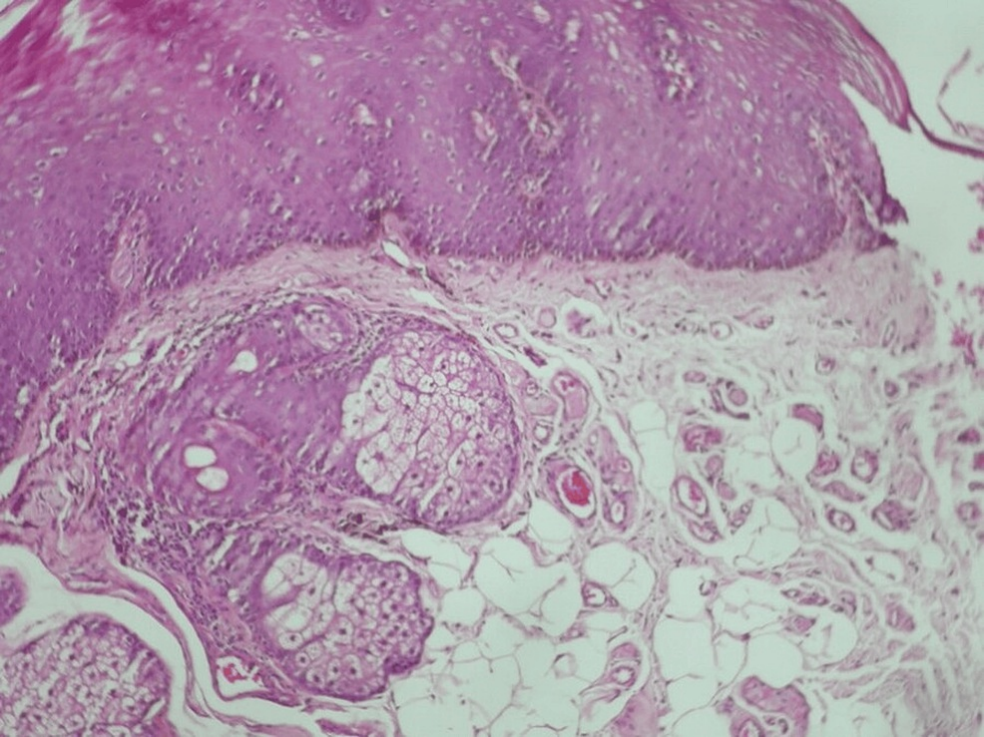
Hyperpigmentation of the basal cell layer of the epithelium

Based on these findings, a biopsy of a melanotic macule of the lip was carried out. The histopathological examination showed that the basal layer of the stratified squamous epithelium exhibited hyperpigmentation (melanin-pigmented basal cells) (Figure 4).

**Figure 5:**
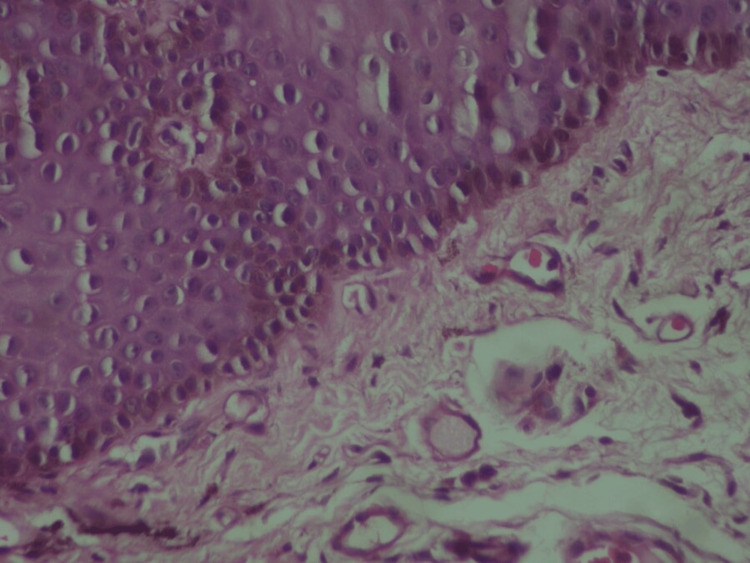
Melaninophages were noted in lamina propria

In addition, scattered melaninophages were noted in lamina propria (Figure 5).

The patient was advised to consult her neurologist, with the purpose of substituting citalopram, the responsible medication. However, the patient reported to be satisfied with the pharmacological effect of her current medication, and thus unwilling to replace it. Therefore, she is going to follow her regular six-month check-up in order to properly photograph and document her oral lesions.

## Discussion

A melanotic macule is a frequent pigmented lesion that affects the oral cavity. The lesion is benign and is caused by an increase in melanin synthesis, often accompanied by an increase in the number of melanocytes [2]. Lesions have been observed in individuals ranging in age from one to 98 years, with an average age of roughly 42 years [15-17]. Lip lesions are typically discovered at a younger age compared to intraoral lesions, presumably due to the fact that it is easier for patients to see and notice abnormalities on the lip's red border (vermillion) as opposed to inside the mouth [18]. Female patients exhibit the highest prevalence of melanotic macules, ranging from 2:1 to 5:1 [15-17,19]. Patients of all races may exhibit melanotic macules and in particular Caucasian patients had a higher prevalence of lower lip lesions, whereas Black patients had a higher incidence of buccal mucosa lesions [16]. Several systemic drugs have been linked to the development of oral and/or cutaneous pigmentation, either by stimulating the production of melanin or by the accumulation of the drug or its byproducts. The medications most commonly associated with this condition include antimalarials, hormones, oral contraceptives, phenothiazines, chemotherapeutics, amiodarone, minocycline, zidovudine, clofazimine, and ketoconazole [3,4,6,20,21]. Patients who are prescribed these drugs for an extended period of time should be closely observed for the occurrence of oral pigmentation [22]. The hard palate, gingiva, and buccal mucosa are the primary sites commonly affected [23]. From a clinical perspective, the discoloration seems even and can be concentrated in one area, multiple areas, or spread out and ranges widely (black, gray, blue, or brown). Pigmentation limited to the hard palate is a characteristic feature associated with antimalarial drugs, which are frequently recommended for the management of rheumatoid arthritis and systemic lupus erythematosus [24]. Minocycline causes blue-black staining of the jawbones in at least 20% of persons who have been using the medicine for over four years [22]. The appearance of bluish tissue on clinical examination may either indicate the darkening of the oral mucosa due to heightened melanin synthesis [20,22,25] or be irrelevant to any genuine pigmentation of the mucosa [6]. The diagnosis relies on establishing a temporal correlation between the occurrence of pigmentation and the commencement of a medicine known to induce this undesirable reaction. Noticeable regression of the lesions may occur following the cessation of the drug [20]. In certain cases, a biopsy may be required to validate the diagnosis, as these discolorations tend to manifest in the same areas where oral melanoma commonly arises [1]. The histopathologic signs of the medication can resemble either melanotic macules or appear as small brown-yellow granules in the lamina propria, which are interpreted as the accumulation of the drug or its byproducts [20].

Psychotropic drugs associated with cutaneous hyperpigmentation include amitriptyline, chlorpromazine, citalopram, desipramine, imipramine, mirtazapine, phenytoin, sertraline, and thioridazine [26]. The hyperpigmentation may initially appear many years after starting the medication. Pathology typically shows melaninophages and melanin in the dermis. Therefore, our case constitutes an exception since citalopram induced intraoral and perioral hyperpigmentation instead of cutaneous; melanin was present in the basal cell layer, and melaninophages in the lamina propria.

## Conclusions

The complete and detailed record of the medical and dental history of the patient may directly unveil the cause of the patient’s symptoms and clinical signs. The prolonged life expectancy is significantly associated with the number of administered drugs. Therefore, the clinician must be on the lookout for drug-induced adverse effects, especially in the elderly, to promptly alleviate or treat.

## References

[1] Rosebush MS, Briody AN, Cordell KG (2019). Black and brown: non-neoplastic pigmentation of the oral mucosa. Head Neck Pathol.

[2] Meleti M, Vescovi P, Mooi WJ, van der Waal I (2008). Pigmented lesions of the oral mucosa and perioral tissues: a flow-chart for the diagnosis and some recommendations for the management. Oral Surg Oral Med Oral Pathol Oral Radiol Endod.

[3] Müller S (2010). Melanin-associated pigmented lesions of the oral mucosa: presentation, differential diagnosis, and treatment. Dermatol Ther.

[4] Eisen D (2000). Disorders of pigmentation in the oral cavity. Clin Dermatol.

[5] Mallikarjuna K, Gupta S, Shukla S, Chaurasia S (2013). Unusual extensive physiologic melanin pigmentation of the oral cavity: a clinical presentation. J Indian Soc Pedod Prev Dent.

[6] Alawi F (2013). Pigmented lesions of the oral cavity: an update. Dent Clin North Am.

[7] Mozaffari HR, Rezaei F, Sharifi R, Mirbahari SG (2016). Seven-year follow-up of Peutz-Jeghers syndrome. Case Rep Dent.

[8] Mignogna MD, Lo Muzio L, Ruoppo E, Errico M, Amato M, Satriano RA (1999). Oral manifestations of idiopathic lenticular mucocutaneous pigmentation (Laugier-Hunziker syndrome): a clinical, histopathological and ultrastructural review of 12 cases. Oral Dis.

[9] Hassona Y, Sawair F, Al-Karadsheh O, Scully C (2016). Prevalence and clinical features of pigmented oral lesions. Int J Dermatol.

[10] Tarakji B, Umair A, Prasad D, Alsakran Altamimi M (2014). Diagnosis of oral pigmentations and malignant transformations. Singapore Dent J.

[11] Lambertini M, Patrizi A, Ravaioli GM, Dika E (2018). Oral pigmentation in physiologic conditions, post-inflammatory affections and systemic diseases. G Ital Dermatol Venereol.

[12] Gondak RO, da Silva-Jorge R, Jorge J, Lopes MA, Vargas PA (2012). Oral pigmented lesions: clinicopathologic features and review of the literature. Med Oral Patol Oral Cir Bucal.

[13] Shulman JD, Beach MM, Rivera-Hidalgo F (2004). The prevalence of oral mucosal lesions in U.S. adults: data from the Third National Health and Nutrition Examination Survey, 1988-1994. J Am Dent Assoc.

[14] Buchner A, Hansen LS (1980). Amalgam pigmentation (amalgam tattoo) of the oral mucosa. A clinicopathologic study of 268 cases. Oral Surg Oral Med Oral Pathol.

[15] Buchner A, Hansen LS (1979). Melanotic macule of the oral mucosa. A clinicopathologic study of 105 cases. Oral Surg Oral Med Oral Pathol.

[16] Kaugars GE, Heise AP, Riley WT, Abbey LM, Svirsky JA (1993). Oral melanotic macules. A review of 353 cases. Oral Surg Oral Med Oral Pathol.

[17] Shen ZY, Liu W, Bao ZX, Zhou ZT, Wang LZ (2011). Oral melanotic macule and primary oral malignant melanoma: epidemiology, location involved, and clinical implications. Oral Surg Oral Med Oral Pathol Oral Radiol Endod.

[18] Buchner A, Merrell PW, Carpenter WM (2004). Relative frequency of solitary melanocytic lesions of the oral mucosa. J Oral Pathol Med.

[19] Tavares TS, Meirelles DP, de Aguiar MC, Caldeira PC (2018). Pigmented lesions of the oral mucosa: a cross-sectional study of 458 histopathological specimens. Oral Dis.

[20] Tosios KI, Kalogirou EM, Sklavounou A (2018). Drug-associated hyperpigmentation of the oral mucosa: report of four cases. Oral Surg Oral Med Oral Pathol Oral Radiol.

[21] Yuan A, Woo SB (2015). Adverse drug events in the oral cavity. Oral Surg Oral Med Oral Pathol Oral Radiol.

[22] Eisen D, Hakim MD (1998). Minocycline-induced pigmentation. Incidence, prevention and management. Drug Saf.

[23] Gaeta GM, Satriano RA, Baroni A (2002). Oral pigmented lesions. Clin Dermatol.

[24] Lerman MA, Karimbux N, Guze KA, Woo SB (2009). Pigmentation of the hard palate. Oral Surg Oral Med Oral Pathol Oral Radiol Endod.

[25] Treister NS, Magalnick D, Woo SB (2004). Oral mucosal pigmentation secondary to minocycline therapy: report of two cases and a review of the literature. Oral Surg Oral Med Oral Pathol Oral Radiol Endod.

[26] Eichenfield DZ, Cohen PR (2016). Amitriptyline-induced cutaneous hyperpigmentation: case report and review of psychotropic drug-associated mucocutaneous hyperpigmentation. Dermatol Online J.

